# Effects of Two Commercial Protein Diets on the Health of Two Imago Ages of *Apis mellifera* L. Reared in Laboratory

**DOI:** 10.3390/ani12080968

**Published:** 2022-04-08

**Authors:** Simona Sagona, Francesca Coppola, Antonio Nanetti, Elena Tafi, Lionella Palego, Laura Betti, Gino Giannaccini, Antonio Felicioli

**Affiliations:** 1 Department of Veterinary Sciences, Pisa University, Viale delle Piagge 2, 56124 Pisa, Italy; simona.sagona@unipi.it (S.S.); francesca.coppola@vet.unipi.it (F.C.); elena.tafi@crea.gov.it (E.T.); 2 Department of Pharmacy, Pisa University, Via Bonanno 6, 56126 Pisa, Italy; laura.betti@unipi.it (L.B.); gino.giannaccini@unipi.it (G.G.); 3 CREA Research Centre for Agriculture and Environment, Via di Saliceto 80, 40128 Bologna, Italy; antonio.nanetti@crea.gov.it; 4 Department of Science, University of the Study of Basilicata, Via dell’Ateneo Lucano 10, 85100 Potenza, Italy; 5 Department of Clinical and Experimental Medicine, Via Savi 10, 56126 Pisa, Italy; lionella.palego@unipi.it

**Keywords:** honey bees, immune system, protein diet, survival, feed intake, health

## Abstract

**Simple Summary:**

Beekeepers often feed their bees with supplemented artificial diets. The formulation of an integrative diet for honey bee colonies able to prevent nutritional deficiencies is yet to be found. In this work, the effects of pollen diet substitution with commercial protein diets in newly emerged bees (that still feed a little on pollen) and in forager bees (that usually do not feed on pollen) were tested. Results obtained suggest that commercial protein diets do not compensate pollen diets in newly emerged bees and do not determine an increase in life span or immunity in forager bees. Further investigations on the effect of concentration and quality of proteins are desirable in order to provide beekeepers with scientific evidence on protein-based feeding.

**Abstract:**

Protein-supplemented artificial diets are widely used by beekeepers during winter and whenever food availability is low, yet no data are available concerning their effects on bees’ health. In this work, the effects of two commercial diets enriched with 1.7% and 7.7% protein concentration on feed intake, survival rate, glucose oxidase, phenoloxidase and glutathione S-transferase in newly emerged and forager bees were tested. Administration of a 7.7% protein-enriched diet significantly reduced the lifespan of both newly emerged and forager bees, while only in foragers a significantly higher feed intake was recorded. In newly emerged bees, administration of a high-protein-enriched diet stimulated glucose oxidase production at the 10th day of feeding, determined a reduction of phenoloxidase and did not affect glutathione S-transferase activity. In forager bees, a high level of protein inclusion did not determine any significant variation in either glucose oxidase, phenoloxidase or glutathione S-transferase activity. Therefore, the results obtained in this investigation suggest that administration of commercial protein diets negatively affect honey bee health, determining an increase in mortality. Further investigations on the effect of concentration and quality of proteins are desirable to provide beekeepers with scientific evidence on protein feeding.

## 1. Introduction

General interest in bee health and nutrition has greatly increased over the last decade [1]. The honey bee immune system is very complex, resulting from both the social and the individual (innate) immune systems [2]. The individual immune system consists of a wide variety of highly effective innate defence barriers based on cellular and humoral responses (i.e., phagocytosis, nodule formation and encapsulation mediated by melanisation) [3,4]. Within individual immune system mechanisms, phenoloxidase (PO) is a key enzyme [5,6], while in the social immune system, the production of the glucose oxidase enzyme appears to play a pivotal role [4]. Glucose oxidase is involved in the conversion of glucose into gluconic acid and hydrogen peroxide, the latter having antibacterial activity [7]. The individual immune system includes the action of haemocytes, antimicrobial peptides and the phenoloxidase enzyme [3]. Phenoloxidase is produced in an inactive form (pro-phenoloxidase), activated through a serine protease and involved in the production of melanin [3]. Melanin is a compound involved in the pathogen encapsulation and the formation of nodules [8]. The antioxidant system also plays a role in the bees’ welfare. Glutathione S-transferase, superoxide dismutase and catalase are honey bee antioxidant enzymes capable of scavenging radical chemical species [9,10,11].

Proteins are a group of macromolecules essential for the honey bee age polyethism [12]. Pollen is the main source of proteins for honey bees, although small percentages of proteins are also present in honey and royal jelly [13]. The protein content of pollen ranges from 2.5 to 61%, based on botanical origin [14,15]. Due to polyethism, a change in the honey bee requirement of pollen occurs [16]. Young adult bees need a larger quantity of pollen, starting to consume it within the first 42–52 h after emergence and achieving the maximum pollen intake at the 8th–9th day of imago life [16]. The pollen intake decreases with age and less pollen utilization was observed in the foragers older than 21 days [17]. The effects of pollen administration to honey bees have been widely investigated. In newly emerged bees, the polyfloral diet induced higher glucose oxidase activity compared to a monofloral supply [18]. In adult queen-less bees, administration of pollen mixtures results in ovary activation [19]. Di Pasquale et al. [20] also observed that when parasitized by *Nosema ceranae*, newly emerged bees fed for 7 days on a polyfloral pollen blend lived longer than bees fed on monofloral pollen. Furthermore, Szymaś and Jędruszuk [21] observed that in newly emerged bees reared for 7–8 days with artificial diets, a lack of protein caused a significant increase in the percentage of granular haemocytes, a significant decrease of other types of haemocytes and lower metabolic activity.

Administration of supplemented artificial diets is a common practice in beekeeping. Sugar- and protein-based artificial diets (i.e., syrup and candy) are especially used during winter, when most of the bees are in an old imago stage and there is a lack of feed (i.e., nectar and pollen) [22].

The effects of sugar and protein supplementation on the honey bees’ immune system have been investigated to assess the role of these macronutrients on bee health at the individual and social level [18,23,24,25,26].

Several studies demonstrated that a protein diet affects honey bee’ physiology [23,27,28,29,30,31]. Camilli et al. [31] observed in reared bees fed with experimental diets enriched with different percentages of crude protein (0, 23, 25 or 27%) that the area and the dimension of the mandibular glands were significantly larger than in the controls. The administration of a protein supplement (MegaBee^®^) resulted in significantly larger acini of hypopharyngeal glands than those belonging to the control group [23]. Several experimental trials proved that protein diet administration in bees determined a shorter lifespan compared to bees fed carbohydrates [28,30]. On the contrary, Zheng et al. [29] reported that a diet with 29.5–34.0% crude protein caused an increase in weight of newly emerged bees, midgut proteolytic enzyme activity, hypopharyngeal gland acini surface and of survival rate.

The formulation of an integrative diet for honey bee colonies able to prevent nutritional deficiencies for particular periods of the year is still to be found. Diets differing in composition, physical appearance and nutrient enrichment have been provided to managed bees to set up an optimal diet [24,32,33,34,35]. In this scenario, this work is aimed to investigate the effect of two commercial protein diets in two imago ages of honey bee survival rate, feed intake, immune system (glucose oxidase and phenoloxidase activities) and antioxidant system (glutathione S-transferase). Newly emerged bees were selected in order to investigate the effects of pollen diet substitution with commercial protein diets, since bees usually feed on pollen from emergence to at least 10 days old [36], while the foragers were selected to test the effect of protein diets in those bees that naturally do not feed on protein but are fed a protein diet in winter as a common beekeeping practice [37]. We predicted that commercial protein diet could compensate for lack of a pollen source in newly emerged bees and improve the health of forager bees.

## 2. Materials and Methods

### 2.1. Honey Bee Sampling and Rearing

Newly emerged (n = 222) and forager (n = 222) honey bees were collected in June 2019 from the experimental apiary of Veterinary Sciences Department of Pisa University, in San Piero a Grado (43°40′51.45″ N 10°20′50.96″ E) from four hives that were previously managed in order to achieve the same family strength (adult/brood), queen age (2 years old), the same treatment against *Varroa* mite (i.e., Oxalic acid in winter) and no evidence of main honey bee disease symptoms such as American foulbrood, deformed wings or diarrhoea. Newly emerged honey bees were sampled with tweezers from frames with brood as they emerged from their cells. Foragers were sampled from the induced bee cluster by previous intentional closing of the hive entrance. For each imago age, 15 bees were collected and stored at −20 °C and were used as T0 in enzymatic assays in absence of feed supplementation. The remaining bees for each imago age were equally distributed in 9 Plexiglass cages (11 cm × 13 cm × 6.5 cm), 3 cages (replicates) per diet, each containing 23 bees. Each cage was equipped with a piece of wax comb and two tip-less syringes (5 mL), one for the diet and one for water [32]. Honey bees were reared at 30 ± 2 °C and natural photoperiod [38]. The cages were grouped according to three commercial diets: diet 1 (with control function), composed of beet sucrose candy with a 0.3% of non-added protein; diet 2 and diet 3, containing beet sucrose enriched with 1.7% and 7.7% of protein from beer yeast (*Saccharomyces cerevisiae* H.), respectively (Table 1). Diet 1 was used as control diet. Protein concentration was determined in accordance with the Bradford method [39] using ovalbumin as reference. Diets and water were administrated *ad libitum* to honey bees and daily renewed.

### 2.2. Survival and Feed Intake

The surviving bees were counted daily throughout the experiment and the dead bees removed from the cages. Survival rate was calculated as percentage of daily survival bees compared to those initially present in cage. Bees used for enzymatic assay were excluded from the count of bees initially present in cage. The feed intake was measured on the same days by weighing each syringe in all diet groups. The daily individual mean consumption was calculated as the ratio between amount of diet consumed and number of living bees.

### 2.3. Enzymatic Assays

All the chemicals used were from Sigma-Aldrich (St. Louis, MO, USA). At day 10 of the feeding period, 5 bees were sampled from each cage and stored at −20 °C until analysis. At day 20, a similar sampling was repeated only from the groups of newly emerged bees.

Glucose oxidase activity was measured on 15 individually analysed bee heads per each diet group [38]. Each sample was weighed before protein extraction; then, 200 µL of 50 mM phosphate buffer pH 7.2 was added. Samples were then incubated at −20 °C for 20 min, homogenized by a Teflon pestle and centrifuged at 1000× *g* at 4 °C for 15 min. The ensuing supernatants were collected. Glucose oxidase was measured accordingly to Cohen Harvey [40], with slight modifications. Prior to analysis, a solution containing 100 mM Hepes buffer pH 7, 0.1 mM EDTA and 5 mM D-glucose was added to the sample. Absorbance data were obtained at λ = 352 nm, at time 0 and 120 min, after the addition of Diaminobenzidine (DAB) (0.18 mg/mL) and HRP (horseradish peroxidase) (0.02 mg/mL). The standard curve was obtained testing different concentrations of H_2_O_2_. Values were expressed as U/mg of tissue, where U indicates the enzymatic unit.

Phenoloxidase and glutathione S-transferase activities were measured on 15 individually analysed bee thoraxes per each diet group [38]. Each sample was weighed before protein extraction and 300 µL of 50 mM phosphate buffer pH 7.2 with 1% (*v*/*v*) Triton X-100 was added. Samples were then incubated at −20 °C for 20 min, homogenized by a Teflon pestle and centrifuged at 1000× *g* at 4 °C for 15 min. The ensuing supernatants were collected, and 200 µL of 50 mM phosphate buffer pH 7.2 was added to pellets and centrifuged at 1000× *g* at 4 °C for 15 min. The supernatants deriving from this second centrifugation were mixed with those previously collected. For the phenoloxidase activity assay, 20 µL of sample was loaded in cuvettes with 505 µL of phosphate saline buffer pH 7.4 and 675 µL MilliQ water, in accordance with Alaux et al. [18]. Cuvettes were incubated at 37 °C for 5 min and 300 µL L-3,4-dihydroxyphenylalanine (L-dopa) (2 mg/mL) was then added. Absorbance data were obtained at λ = 490 nm, at time 0 and 10 min. Values were expressed as mU/mg of tissue.

Glutathione S-transferase activity was analysed using a slightly modified procedure from Habig et al. [41]. In more detail, a solution containing 1.23 mL 25 mM phosphate buffer pH 6.5, 50 µL 1 mM 1-chloro-2,4-dinitrobenzene (CDNB) in methanol, 200 µL MilliQ water and 50 µL 5 mM GSH (glutathione reduced) was first incubated at 30 °C for 5 min. Before the absorbance measure, 20 µL of sample was added to this solution. Absorbance data were obtained at λ = 340 nm at time 0 and 5 min. Values were expressed as mM/min/mg of tissue, where the unit of measure mM refers to the concentration of the product resulted from the conjugation of CDNB with reduced glutathione. Enzymatic assays were performed by the Lambda 25 UV/VIS spectrophotometer (Perkin Elmer, Milan, Italy).

### 2.4. Statistical Analysis

For inferential statistics, the estimated survival rate was analysed by the Wilcoxon rank test, using the product–limit (Kaplan–Meier) method. The sum of daily survival bees in each replicate for each diet was used to test statistically significant differences in survival rate among diets. Statistics for feed intake, glucose oxidase, phenoloxidase and glutathione S-transferase activities were performed as follows: data residues, obtained by preliminary ANOVA for more factors, were tested for normal distribution by means of the Shapiro–Wilk test. Since data resulted not normally distributed, the effect of diet was statistically analysed by a non-parametric Kruskal–Wallis H-test, followed by post hoc Mann–Whitney U-test pairwise comparisons. All statistic calculations were performed using JMP software (SAS Institute, Cary, NC, USA, 2008). The 2-tailed α-error was pre-set at 0.05.

## 3. Results

### 3.1. Protein Content, Feed Intake and Survival

**Figure 1:**
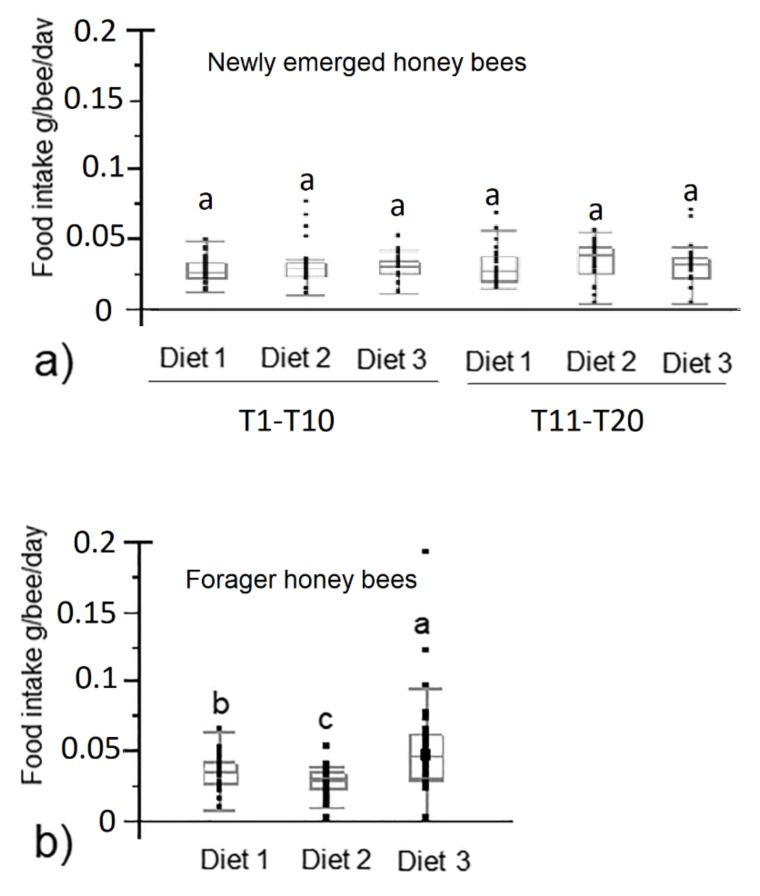
Feed intake of newly emerged from T1 to T10 and from T11 to T20 (**a**) and forager honey bees (**b**) fed with control diet and experimental diets (diet 1, diet 2, diet 3 with 0.3%, 1.7% and 7.7% of protein content, respectively). Different letters indicate statistical significance (*p* < 0.01) by Kruskal–Wallis test.

**Figure 2:**
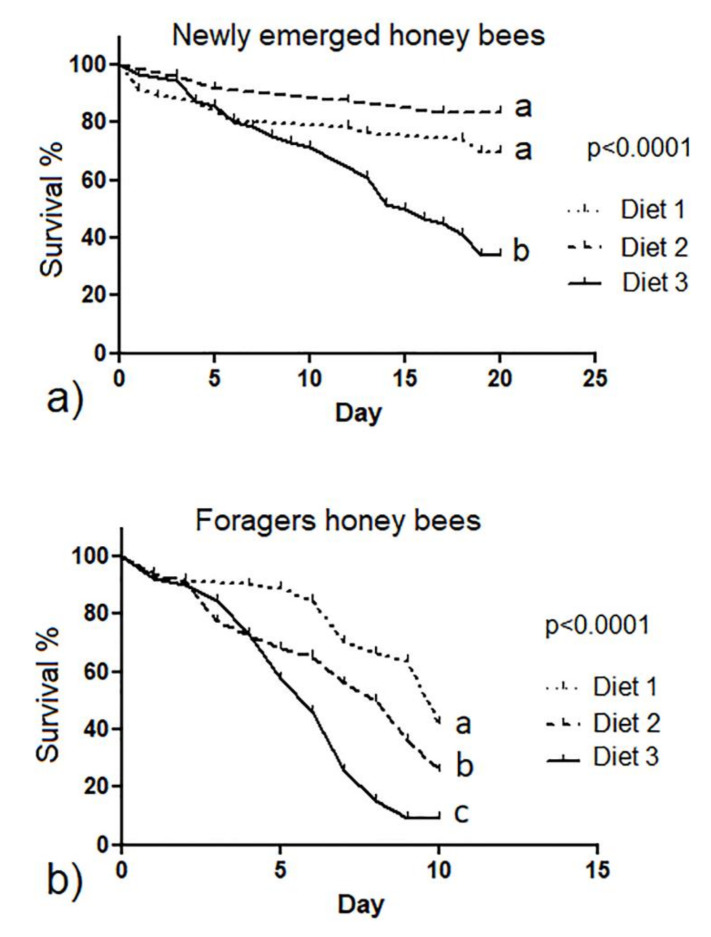
Survival rate of newly emerged (**a**) and forager honey bees (**b**) fed with control and experimental diets (diet 1, diet 2 and diet 3 with 0.3%, 1.7% and 7.7% of protein content, respectively). Different letters indicate statistical significance (*p* < 0.0001) by Kaplan–Meier test.

According to the results of the Bradford assay, the content of total protein of diet 2 and 3 was 1.7% and 7.7%, respectively. The Bradford assay also showed that the content of protein in the control diet 1 was 0.3%. Feed intake in newly emerged honey bees did not statistically differ among the three diet groups (*p* > 0.05) (Figure 1a). Forager bees belonging to the diet 3 group consumed significantly more feed than those belonging to the diet 1 and diet 2 groups (*p* < 0.01) (Figure 1b). Forager honey bees belonging to the diet 2 group consumed significantly less feed (median 0.029 g/bee/day) than those belonging to the other two diet groups (median 0.034 and 0.047 g/bee/day for diet 1 and diet 3, respectively; *p* < 0.01). The survival rate in newly emerged honey bees was significantly higher in honey bees fed on diet 1 and diet 2 compared to those fed on diet 3 (*p* < 0.0001) (Figure 2a). The survival rate in foragers was significantly higher in the diet 1 group than in the other two diet groups (*p* < 0.0001) (Figure 2b).

### 3.2. Glucose Oxidase Activity

**Figure 3:**
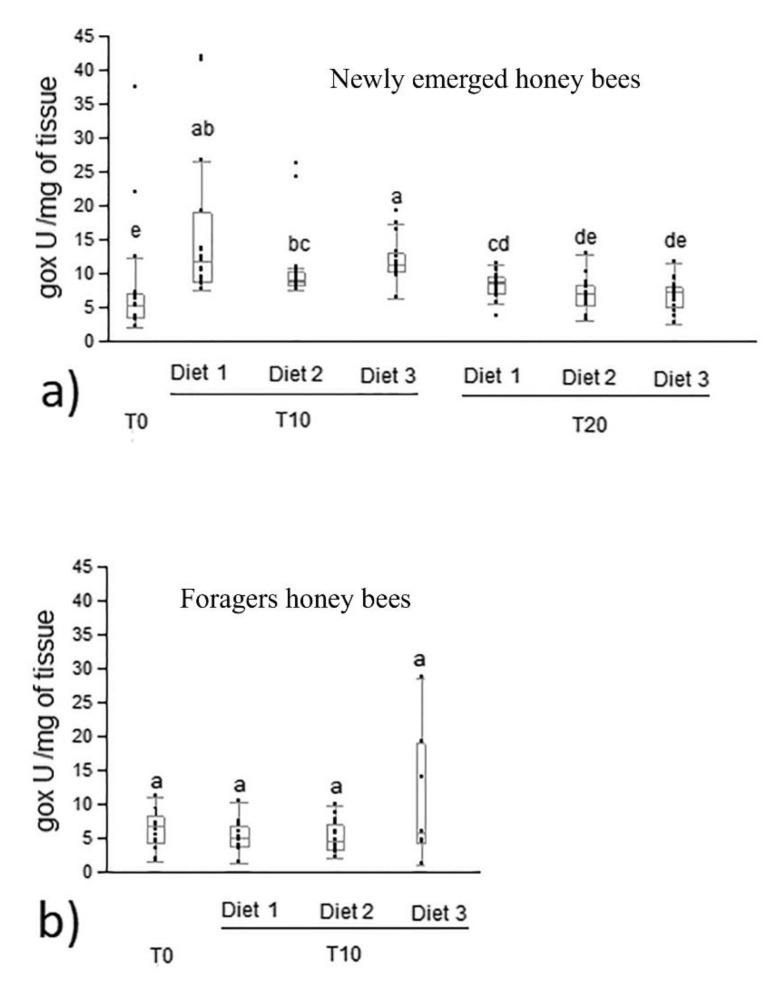
Glucose oxidase activity in newly emerged (**a**) and forager (**b**) honey bees fed with control and experimental diets (diet 1, diet 2, diet 3 with 0.3%, 1.7% and 7.7% of protein content, respectively). Different letters on each bar indicate statistical significance (*p* < 0.0001) by Kruskal–Wallis test. Data were expressed as U/mg of tissue (median, min and max values). T0, T10, T20 were the day of honey bee sampling, n = 15 per bar.

Newly emerged honey bees belonging to all diet groups showed an increase of glucose oxidase activity at day 10 of the feeding period compared to day 0 (Figure 3a). At the 10th day of feeding, newly emerged honey bees belonging to the diet 3 group showed a significantly higher glucose oxidase activity than those belonging to diet 2 (median 11.26 and 8.91 U/mg of tissue, respectively; *p* < 0.0001). No differences were recorded in newly emerged bees between diet 1 and diet 2 (*p* > 0.05). At the 20th day of feeding, glucose oxidase activity was higher in newly emerged honey bees fed with diet 1 (median 8.52 U/mg of tissue) than in those collected at time 0 (5.15 U/mg of tissue). No differences were recorded on glucose oxidase activity among newly emerged honey bees fed with diet 1, diet 2 and diet 3. In forager honey bees, no differences in glucose oxidase activity were recorded between time 0 and at the 10th day of feeding, as well as among the three forager diet groups at day 10 (Figure 3b).

### 3.3. Phenoloxidase Activity

**Figure 4:**
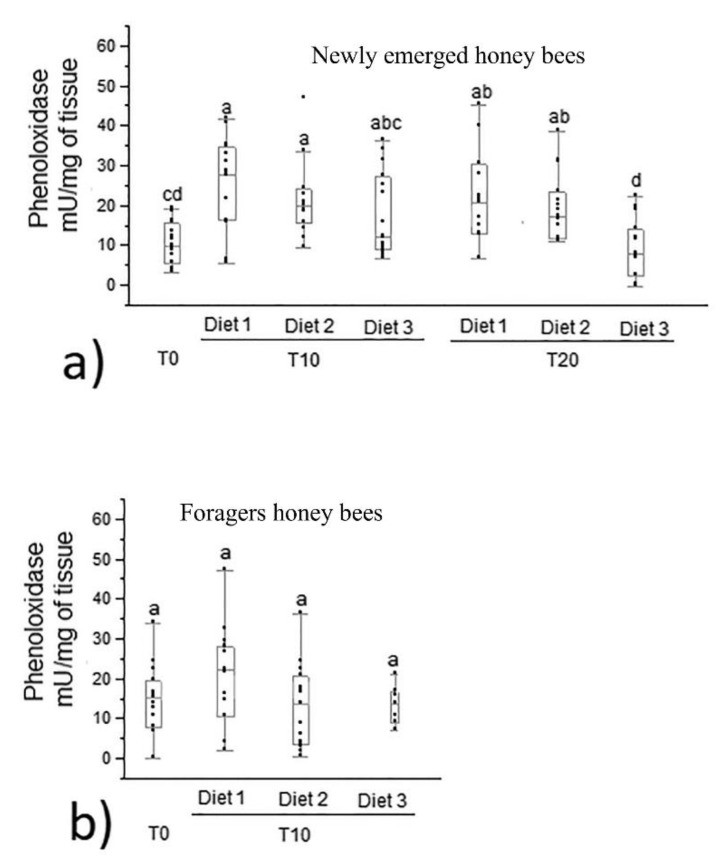
Phenoloxidase activity in newly emerged (**a**) and forager (**b**) honey bees fed control and experimental diets (diet 1, diet 2, diet 3 with 0.3%, 1.7% and 7.7% of protein content, respectively). Different letters indicate statistical significance (*p* < 0.0001) by Kruskal–Wallis test. Data were expressed as mU/mg of tissue (median, min and max values). T0, T10, T20 were the days of honey bees sampling, n = 15 per bar.

Phenoloxidase activity in newly emerged honey bees significantly increased in diet 1 and diet 2 groups (median 27.75 and 19.84 mU/mg of tissue, respectively) at the 10th day of feeding compared to time 0 (median 9.70 mU/mg of tissue; *p* = 0.0002). No statistical differences were recorded in phenoloxidase activity in newly emerged bees fed diet 3 (*p* > 0.05) (Figure 4a). At the 10th day of feeding, no statistical differences (*p* > 0.05) were recorded in phenoloxidase activity among the three diet groups (diet 1, diet 2 and diet 3). At the 20th day of feeding, phenoloxidase activity was significantly higher in newly emerged honey bees fed with both diet 1 and diet 2 (median 20.83 and 17.31 mU/mg of tissue, respectively) than in those belonging to the diet 3 group (median 7.94 mU/mg of tissue; *p* < 0.0001). At the 20th day of feeding, phenoloxidase activity in groups fed diet 1 and diet 2 did not show statistical differences compared to the 10th day, while it appeared significantly lower in honey bees fed diet 3. Phenoloxidase activity in newly emerged honey bees belonging to the diet 3 group at the 20th day did not show statistical differences to bees sampled at time 0. No significant differences were recorded between T0 and the experimental diet groups (diet 2 and 3) and the control one (diet 1) at the 10th day of feeding (*p* > 0.05) (Figure 4b).

### 3.4. Glutathione S-transferase Activity

**Figure 5:**
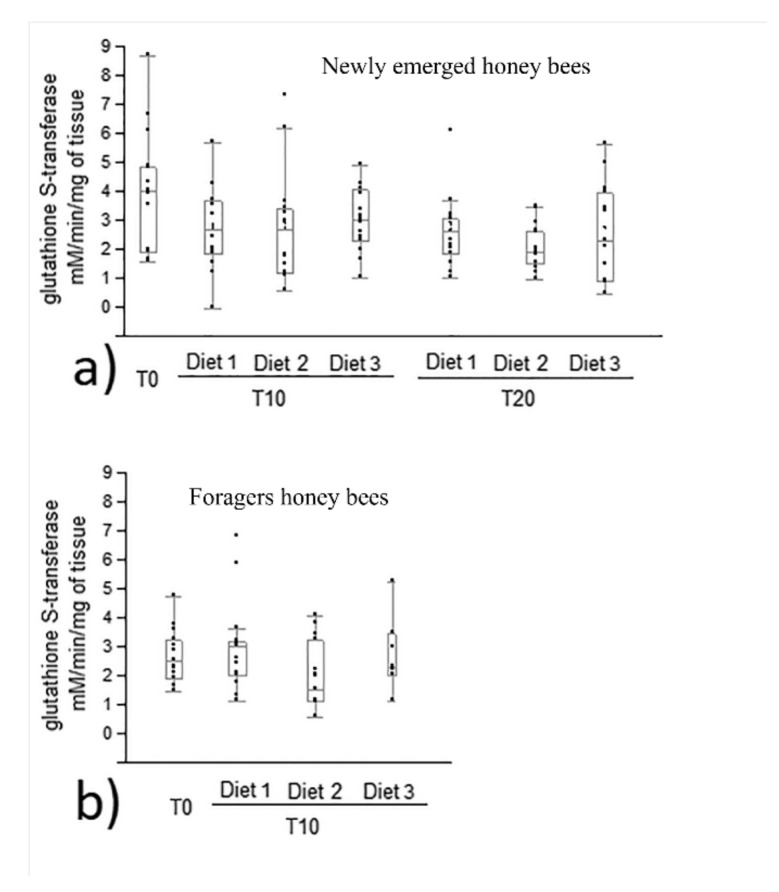
Glutathione S-transferase activity in newly emerged (**a**) and forager (**b**) honey bees fed control and experimental diets (diet 1, diet 2, diet 3 with 0.3%, 1.7% and 7.7% of protein content, respectively). No statistical differences were observed by Kruskal–Wallis test (*p* > 0.05). Glutathione S-transferase activity was expressed as mM/min/mg of tissue (median, min and max values). T0, T10, T20 were the days of honey bee sampling, n = 15 per bar.

In both newly emerged and forager honey bees, no diet group showed statistical differences in glutathione S-transferase activity at the 10th day of feeding compared to time 0 (Figure 5a,b). In addition, no statistical differences were recorded among each diet group in newly emerged bees in glutathione S-transferase activity at the 10th and 20th day of feeding (*p* = 0.087, Figure 5a).

## 4. Discussion

Results obtained in this investigation show that feed intake was significantly higher in forager bees fed with the high-protein diet (diet 3; 7.7% crude protein content) than other diet groups, while no differences were recorded between diet groups in newly emerged bees. The high feed intake recorded in forager bees fed the high-protein diet could be the result of several factors, such as the higher quality of protein in diet 3 compared to the other two diets leading to an increase of feed palatability, as previously hypothesized by Noordyke and Ellis [42]. In contrast, it may be that bees preferred the high-concentration protein diet driving from the inferior quality of the low-protein concentration diet [42]. In addition, it cannot be excluded that bees consumed a considerably higher quantity of the high-protein concentration diet than the other two diets to fulfil their nutritional needs [42]. The protein requirement is high in newly emerged bees until 10 days of imaginal life for hypopharyngeal gland development, and after this period bees are no longer able to metabolize high amounts of proteins [43]. Moreover, according to Zheng et al. [29], the protein excess in honey bee diets could determine the accumulation of indigested material in the gut and the extra proteins could inhibit the absorption of other substances, resulting in a shorter lifespan. Results obtained in this study also show that the high-protein-enriched diet determined a significant reduction of lifespan in both newly emerged and forager bees, thereby the experiment with forager bees was stopped at the 10th day due to high bee mortality. These results are in accordance with Lamontagne-Drolet et al. [30], who observed that honey bees fed with a protein-enriched diet (Global Patties^®^ and Ultra Bee^®^ with 17% and 18% crude protein content, respectively) showed a significantly shorter lifespan than control bees. Reduction of survival was also observed in bees fed with rape-pollen-based diets supplemented with different crude protein levels (25.0, 29.5, 34.0 and 38.5%) [29]. Therefore, the reduction of lifespan recorded in forager honey bees in this investigation could likely be due to a high intake of low digestibility protein as well as to an imbalance of the protein–fat ratio in the diet.

Administration of protein-enriched diets determined an increase of glucose oxidase activity in newly emerged bees at the 10th day of rearing. An increase of glucose oxidase was also reported by Alaux et al. [18] and by Takenaka et al. [44] in newly emerged bees fed with a pollen diet administered for 10 days, compared to a candy diet. Glucose oxidase is expressed in the hypopharyngeal glands of worker bees [44] and an increase of the acini size of hypopharyngeal glands was observed in newly emerged bees fed pollen for 7 days, compared to bees reared with a no pollen diet [20]. Therefore, the increase of glucose oxidase recorded in newly emerged bees could be due to a supplemented protein-dependent increase of the hypopharyngeal glands. However, glucose oxidase activity in newly emerged bees fed protein-enriched diets significantly decreased at the 20th day to levels non-significantly different to those recorded at T0. The reduction of glucose oxidase activity recorded in this study in newly emerged bees recorded at the 20th day contrasts with the physiological conditions occurring in free-flying colonies, where an increased glucose oxidase activity was observed in workers of roughly 23 days of age [44]. Therefore, the reduction of glucose oxidase production in the long term recorded in newly emerged bees in this study could be due to the absence of physiological stimuli such as the absence of queen, brood, lack of bee handling of bee products (such as wax, propolis, bee pollen) and trophallaxis. According to Takenaka et al. [44], no differences in glucose oxidase activity were recorded between forager bee diet groups at the 10th day.

In this investigation, phenoloxidase activity in newly emerged bees significantly increased in diet 1 and diet 2 groups at the 10th day of feeding compared to time 0. In the diet 3 group (high protein content), PO activity slightly increased at the 10th day, but was not statistically different from both T0 and the other two diet groups. According to Schmid et al. [3], PO activity in honey bees reaches a plateau within the first week of adult life. At the 20th day of feeding, in newly emerged bees fed with the high-protein-enriched diet (diet 3), phenoloxidase was significantly lower than that recorded at the 10th day and did not significantly differ from T0. Therefore, the result obtained in this investigation suggests that administration of a high-protein-enriched diet in newly emerged bees inhibits phenoloxidase activity. In addition, the same diet group at the 20th day also showed the highest mortality rate, which suggests that bee mortality could be linked to a low basal activity of phenoloxidase. This result is in accordance with the hypothesis that bee survival is positively linked to a high basal activity of phenoloxidase [20,38,45]. Therefore, the intake of an excess of proteins could affect any point in the enzyme cascade or even its regulation; therefore, further investigations to clarify the mechanism linking the protein diet concentration to the decrease of phenoloxidase are needed.

In forager bees, no differences were recorded in phenoloxidase activity between those fed with protein-enriched diets and the control ones. The result obtained in this study indicates that protein level in diet, at least at this concentration, did not stimulate phenoloxidase activity as previously reported by Alaux et al. [18].

Glutathione S-transferase activity did not show differences between diet groups both in newly emerged and forager bees, suggesting that protein level in the bee diet did not affect glutathione S-transferase activity. An increase of glutathione S-transferase in newly emerged and forager bees was previously reported by Di Pasquale et al. [20] as an effect of several types of pollens, suggesting that proteins may influence the glutathione S-transferase activity. Therefore, the absence of a significant protein effect on glutathione S-transferase activity in this study may be due to either concentration or quality of the proteins consumed by experimental bees.

## 5. Conclusions

In conclusion, protein supplementations in newly emerged bees did not affect feed intake and significantly reduced the life span. Administration of a high-protein diet stimulated glucose oxidase production at the 10th day of feeding, determining a reduction of phenoloxidase activity and not affecting glutathione S-transferase. In forager bees, a high level of protein inclusion significantly increases feed intake and reduces bee survival rate, while not determining any significant variation in glucose oxidase, phenoloxidase and glutathione S-transferase activity. Therefore, the results obtained in this investigation indicate that administration of commercial high-protein diets are not able to compensate a lack of pollen source in newly emerged bees and negatively affect the health of forager bees, determining an increase of mortality. Further investigation on the effect of adding different concentrations and qualities of proteins are desirable to provide beekeepers with scientific evidence concerning the use of protein feeding for bees.

## Figures and Tables

**Table 1:** Composition of the three experimental diets (diet 1, diet 2, diet 3) reported on commercial labels.

	Diet 1	Diet 2	Diet 3
**General composition**	Sugar, glucose syrup, sterilized water	Beet sugar, glucose syrup, water, beer yeast extract, proteins	Sucrose, glucose/fructose syrup, water, beer yeast extract, proteins
**Analytical components**			
Carbohydrates	-	92%	85%
sugars	-	92%	81%
Crude protein	-	<0.1%	6.2%
Crude ash	-	<0.1%	0.76%
Crude fats	-	<0.1%	0.36%
**Hydroxymethylfurfural (HMF)**	<4 mg/kg	<2 mg/kg	<1.25 mg/kg
**Humidity**	-	-	<8.5%

## Data Availability

All data are available from the corresponding author.
